# Supplementary material for: RBM20 Regulates CaV1.2 Surface Expression by Promoting Exon 9* Inclusion of CACNA1C in Neonatal Rat Cardiomyocytes
Source: Int J Mol Sci. 2019 Nov 8;20(22):5591. doi: 10.3390/ijms20225591 (PMC6888234; doi:10.3390/ijms20225591)
Supplement: Supplementary file 1 [file ijms-20-05591-s001.pdf]

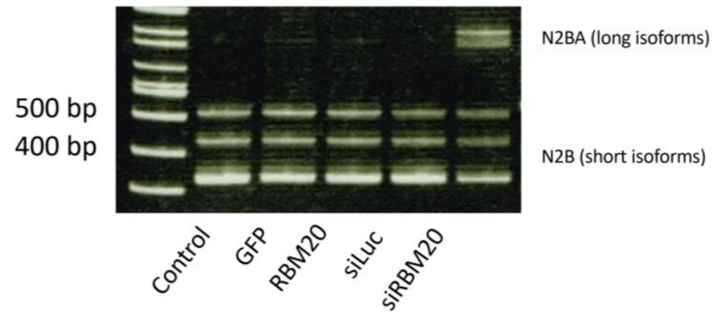

Figure S1. Titin splice variant expression upon RBM20 overexpression or siRNA knockdown. Titin splice isoform mRNA expression was measured by RT-PCR in control, GFP, RBM20-overexpressing (RBM20), luciferase-1-targeting siRNA (siLuc), or RBM20 siRNA (siRBM20) conditions. The picture is representative of three experiments from four cardiomyocyte preparations.

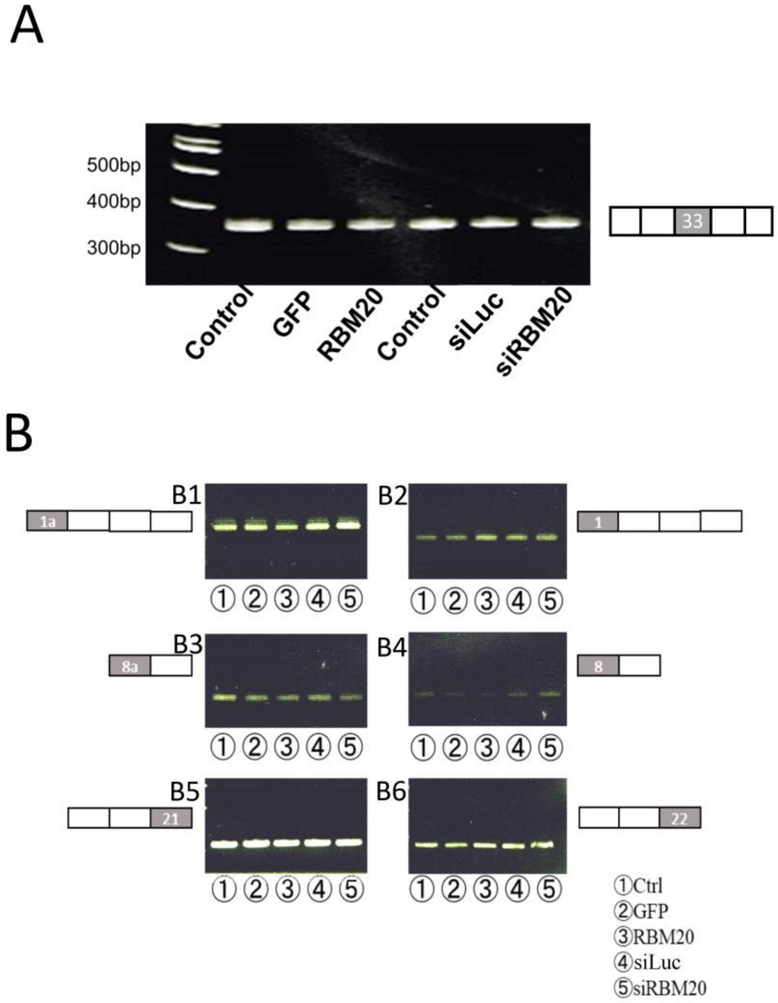

Figure S2. RBM20 does not regulate exon 33 alternative splicing or mutually exclusive exons. (A) The picture shows the results of the RT-PCR experiments showing the amplicon from the CaV1.2 exon 30–35 region in control, GFP, RBM20-overexpressing (RBM20), luciferase-1-targeting siRNA (siLuc), or RBM20 siRNA (siRBM20) conditions. The picture is representative of n = 4 experiments from four cardiomyocytes preparations. (B) The pictures display the representative results of the RT-PCR experiments, showing amplicons of regions covering exons 1a–4 (B1); 1–4 (B2); 8a–9 (B3); 8–9 (B4); 19–

21 (B5); and 19, 20, and 22 (B6) in control, GFP, RBM20-overexpressing (RBM20), luciferase-1-targeting siRNA (siLuc), or RBM20 siRNA (siRBM20) conditions. The picture is representative of n = 3 experiments from three cardiomyocyte preparations.
